# Whole-Genome Sequences and Classification of *Streptococcus agalactiae* Strains Isolated from Laboratory-Reared Long-Evans Rats (*Rattus norvegicus*)

**DOI:** 10.1128/genomeA.01435-16

**Published:** 2017-01-05

**Authors:** C. Bodi Winn, J. Dzink-Fox, Y. Feng, Z. Shen, V. Bakthavatchalu, J. G. Fox

**Affiliations:** Division of Comparative Medicine, Massachusetts Institute of Technology, Cambridge, Massachusetts, USA

## Abstract

In collaboration with the CDC’s *Streptococcus* Laboratory, we report here the whole-genome sequences of seven *Streptococcus agalactiae* bacteria isolated from laboratory-reared Long-Evans rats. Four of the *S. agalactiae* isolates were associated with morbidity accompanied by endocarditis, metritis, and fatal septicemia, providing an opportunity for comparative genomic analysis of this opportunistic pathogen.

## GENOME ANNOUNCEMENT

*Streptococcus agalactiae*, or group B streptococcus (GBS), is a Gram-positive opportunistic pathogen that commonly inhabits the gastrointestinal and urogenital tract of humans. It is the leading cause of neonatal septicemia and meningitis, and it is also known to cause disease in animals, including meningoencephalitis in fish, mastitis in cattle, and invasive infection in laboratory rodents (1–6). There is high suspicion that GBS is a zoonotic pathogen, and despite close genotypic relationships observed between different species’ isolates, direct transmission has not been reported (7–9). In this report, we announce the whole-genome sequences of seven GBS isolates obtained from a colony of laboratory-reared Long-Evans rats that experienced two cases of polymicrobial sepsis associated with GBS infection in two gravid female rats; two isolates were obtained from resected cardiac tissue and blood in the first case, and two isolates were obtained from cardiac tissue and fetus from the second case. An additional three asymptomatic male rats’ GBS isolates were cultured from the nares. Isolates were 16S rRNA sequenced and submitted to the CDC’s *Streptococcus* Laboratory for whole-genome sequencing.

The CDC’s *Streptococcus* Laboratory tracks cases of GBS invasive disease through their Active Bacterial Core surveillance (ABCs). After receipt of isolates, genomic DNA for short-read whole-genome sequencing was extracted manually using a modified QIAamp DNA minikit protocol (Qiagen). Nucleic acid concentration was quantified by an Invitrogen Qubit assay, and samples were sheared using a Covaris M220 ultrasonicator programmed to generate 500-bp fragments. Libraries were constructed using TruSeq DNA PCR-free high-throughput (HT) library preparation kit with 96 dual indexes and quantified by a Kapa quantitative PCR (qPCR) library quantification method. Genomes were sequenced by Illumina NGS technology using MiSeq version 2 500-cycle kit. Genomes were annotated using Rapid Annotations using Technology toolkit (RASTtk) (10, 11) (http://rast.nmpdr.org/rast.cgi).

Strains underwent the following analyses: (i) multilocus sequence typing (MLST; http://pubmlst.org/sagalactiae) (12), (ii) serotyping (13), (iii) detection of resistance genes with ResFinder (14) and the Comprehensive Antibiotic Resistance Database (CARD) (15), (iv) virulence factors (VFDB; http://www.mgc.ac.cn/VFs/) (16), and (v) antimicrobial susceptibility testing by a microdilution broth method.

MLST analysis and serotyping assigned all GBS strains except strain 195-16-B-RAT to serotype Ib, sequence type 12 (ST12) (13, 17). Strain 195-16-B-RAT is serotype V, ST1. Both serotype-sequence types are well-represented among human invasive isolates documented in the 2015 ABCs (unpublished data). Isolates were susceptible to ampicillin (≤0.25 μg/ml), ceftriaxone (≤0.5 μg/ml), erythromycin (≤0.25 μg/ml), and clindamycin (≤0.25 μg/ml) but resistant to tetracycline (>8 μg/ml). Strains were intermediate resistant to enrofloxacin (≤0.5 μg/ml), a commonly used fluoroquinolone in veterinary medicine (18, 19). Strain 195-16-B-RAT had a mutation detected in the *ermA* gene, denoting resistance to erythromycin. This may be due to inducible expression of the resistance-causing methylase enzyme, which leads to different phenotypes (20). All isolates had *tet*(M), associated with tetracycline resistance. The virulence factors identified were β-hemolysin/cytolysin (*cylE*), CAMP factor (*cfa/cfb*), hyaluronate lyase (*hylB*), C5a peptidase (*scpB*), pili (*pilA, pilB, pilC*), sortases (*srtC3, srtC4*), and Fg-binding surface protein A and B (*fbsA* and* fbsB*).

### Accession number(s).

The whole-genome sequences of the seven *S. agalactiae* strains have been deposited at DDBL/EMBL/GenBank under the accession numbers provided in Table 1.

**TABLE 1:** Genomic characteristics of rat *Streptococcus agalactiae* strains

*S. agalactiae* strain	GenBank accession no.	Origin of rat isolates	Presence of clinical disease	Genome size (bp)	No. of contigs	Fold coverage	G+C content (%)	No. of protein-coding sequences	No. of RNA sequences
195-16-B-RAT	MJHP00000000	Nares	No	2,134,553	22	50	35.2	2,146	56
196-16-B-RAT	MJHQ00000000	Blood	Yes	2,060,190	41	50	35.1	2,064	61
197-16-B-RAT	MJHR00000000	Cardiac lesion	Yes	2,065,096	35	50	35.1	2,072	61
198-16-B-RAT	MJHS00000000	Nares	No	2,060,782	38	50	35.1	2,078	65
199-16-B-RAT	MJHT00000000	Nares	No	2,074,289	37	50	35.1	2,080	68
200-16-B-RAT	MJHU00000000	Cardiac lesion	Yes	2,068,751	37	100	35.1	2,084	76
201-16-B-RAT	MJHV00000000	Fetus	Yes	2,063,718	35	100	35.1	2,082	70
